# Mesenchymal stem cells for recurrent miscarriage

**DOI:** 10.1186/s12967-026-08179-x

**Published:** 2026-04-24

**Authors:** Famela S. Ramos, Jesus Perez, George K. Ng, James D. Veltmeyer, Michael P. Koumjian, Feng Lin, Dede Byrne, George Delgado

**Affiliations:** 1 Aureum Therapeutics Inc, Chula Vista, CA US; 2 Loma Linda University, Loma Linda, CA US; 3 Little Workers of the Sacred Hearts, Baltimore, US; 4 Steno Institute, San Diego, CA US

## Abstract

**Background:**

Recurrent pregnancy loss (RPL), affecting 1–5% of couples, is frequently driven by immunological dysregulation, including excessive natural killer (NK) cell cytotoxicity, reduced regulatory T cell (Treg) function, Th1/Th17 dominance, and inflammatory cytokine imbalance at the maternal-fetal interface. These abnormalities disrupt immune tolerance to the semi-allogeneic fetus, leading to implantation failure or early miscarriage. Current interventions (e.g. low-dose aspirin/heparin for antiphospholipid syndrome, intravenous immunoglobulin, or corticosteroids) show variable efficacy and limitations, particularly in unexplained cases. Mesenchymal stem cells (MSCs), with proven immunomodulatory, anti-inflammatory, and tissue-repair properties, have gained regulatory approval for severe immune dysregulation conditions like steroid-refractory graft-versus-host disease (GVHD), which shares pathophysiological parallels with immune-mediated RPL, including NK hyperactivity and deficient Treg tolerance.

**Main body:**

MSCs from sources such as bone marrow, adipose tissue, or umbilical cord exert context-dependent effects, secreting anti-inflammatory factors (e.g. IL-10, TGF-β), suppressing pro-inflammatory cytokines, inhibiting cytotoxic NK activity, and promoting Treg expansion and function to restore immune homeostasis. Preclinical studies in abortion-prone mouse models (e.g. CBA/J × DBA/2) demonstrate that MSCs reduce fetal resorption by attenuating inflammation, enhancing placental angiogenesis, and balancing decidual immune responses. Emerging human data support MSCs for uterine repair in conditions like thin endometrium or Asherman syndrome, improving endometrial thickness and pregnancy outcomes. Unlike broad immunosuppressants, MSCs offer targeted, responsive modulation with low immunogenicity, minimal tumorigenicity, and potential for autologous or allogeneic use via simple infusion routes.

**Conclusion:**

MSCs represent a promising, novel therapeutic strategy for immunologically mediated RPL by addressing core immune imbalances and supporting placental/tissue health, potentially outperforming existing options in unexplained cases. Building on FDA-approved MSC therapies for analogous immune pathologies and encouraging preclinical results, clinical investigation of MSC-based interventions is warranted to establish safety, efficacy, and optimal protocols for this challenging condition.

**Clinical trial number:**

Not applicable.

## Introduction

Pregnancy is a unique immunological paradox where the maternal immune system tolerates a semi-allogeneic fetus while maintaining defenses against pathogens [1, 2]. The phenomenon of pregnancy is not explained by immunological ignorance or anatomical sequestration of immune cells because studies have shown maternal cells enter the developing fetus and fetal cells enter the mother, in some situations remaining in the allogeneic environment for years [3–5]. Pregnancy thus is a state of “temporary immunological reprogramming.” In a successful pregnancy, immune tolerance is established at the maternal-fetal interface, involving a delicate balance of pro- and anti-inflammatory responses. This tolerance is mediated by specialized immune cells, cytokines, and regulatory mechanisms that prevent rejection of the fetus without systemically immunologically compromising the mother.

When the delicate “tolerogenic balance” is disrupted, pregnancy loss can occur, usually before 20 weeks of gestation [6]. Recurrent miscarriage (RM), or recurrent pregnancy loss (RPL), affects 1–5% of couples and is usually defined as two or more consecutive losses. Up to 50–60% of RM cases are attributed to immunological factors, including autoimmune and alloimmune abnormalities [7].

The detailed mechanisms of successful pregnancy involve a shift towards reduced T cell immunity to fetal antigens [8], and increased regulatory T cells (Tregs) and tolerogenic NK cells at the decidua [9–11], promoting anti-inflammatory cytokines like IL-10 and TGF-β [12]. IL-10 dampens inflammatory immunity by suppression of antigen presentation and depression of inflammatory cytokine production [13] while TGF- β protects pregnancy by the stimulation of Treg cells as well as reducing macrophage activation [14].

Abnormal immune responses in miscarriages are characterized by excessive T helper type 1 and T helper type 17 (Th1/Th17)activity [15, 16], heightened NK cell cytotoxicity [17, 18], and inflammatory cytokine production [19–21], leading to coagulopathy and fetal loss [22]. The clinical relevance of these immunological abnormalities is suggested by studies in which pregnancy rate is increased after intervention. For example, TNF-alpha blockade has been shown to increase successful pregnancy in woman with RPL in a placebo-controlled trial [23]. Support for an immunological basis for RPL also comes from the animal model of this condition, the CBA/J female ×DBA/2 male mouse cross in which fetal loss is associated with similar mechanisms that occur in the clinical situation [24]. Blockade of inflammatory cytokines such as IL-17 [25, 26], and administration of anti-inflammatory cytokines such as IL-10 [27, 28], IL-1 receptor antagonist [29], and soluble HLA-G [30], preserves pregnancy in this model. Interestingly, suppressors of complement activation also have pregnancy promoting properties [31–33].

Mesenchymal stem cells (MSC) are multipotent adult stem cells found in various tissues, such as bone marrow, adipose tissue, umbilical cord, and amniotic fluid, characterized by their ability to self-renew and differentiate into multiple cell types, including osteoblasts, chondrocytes, and adipocytes [34, 35]. These cells play a critical role in tissue repair and regeneration due to their immunomodulatory, anti-inflammatory, and trophic properties, which allow them to regulate immune responses and support tissue healing. MSCs are easily isolated and expanded in culture, making them a promising tool in regenerative medicine and research, particularly for conditions involving inflammation, tissue damage, or immune dysregulation, or autoimmune disorders [36].

MSC have an extensive clinical history with positive therapeutic signals seen in clinical trials of major degenerative, autoimmune, and inflammatory diseases including liver failure [37–39], heart failure [40–52], multiple sclerosis [53–57], rheumatoid arthritis [58–60], type 1 diabetes [61–63], osteoarthritis [64, 65], and in some cases even aging itself [66].

Given the ability of MSC to produce immune regulatory and complement inhibitory factors [67], as well has their demonstrated ability to suppress even extreme cytokine/inflammatory related pathologies such as COVID-19 [68, 69], sepsis [70], and GVHD [71], we propose investigation of MSC for “dampening” inflammatory predispositions in RPL and allowing for non-pathologic maternal immune regulatory mechanisms to predominate. Mechanistically, MSC induce a self-perpetuating cycle of tolerogenesis in part through stimulation of Treg cell production and activity. Stimulation of Treg by MSC is believed to occur by different pathways including MSC production of TGF-beta and IL-10 [72, 73], as well as expression of indolamine 2,3 dioxygenase [74]. Additionally, MSC can induce regulatory dendritic cells [75, 76], which are capable of stimulating Treg generation, which eventually induces the tolerogenic loop discovered by Min et al. [77].

In this paper we synthesize current evidence on these mechanisms, emphasizing the roles of key immune components and their implications for RPL and then we discuss how MSC therapy may be useful in remedying these abnormalities.

## Normal immune mechanisms in pregnancy

To understand abnormalities, it is essential to outline normal immunological processes associated with pregnancy. Implantation begins with the blastocyst’s apposition and adhesion to the receptive endometrium, mediated by molecular interactions such as integrins and selectins [78]. As the blastocyst embeds, the trophoblast differentiates into two layers, with cytotrophoblasts forming the inner layer and syncytiotrophoblasts, creating a multinucleated outer barrier. Extravillous trophoblasts (EVT) detach from the distal trophoblast cell columns and invade the decidua, remodeling maternal spiral arteries to establish low-resistance blood flow critical for fetal nourishment and growth [79].

Uterine NK (uNK) cells, characterized as CD56+ bright CD16-, dominate the decidual immune landscape during early pregnancy, particularly during the implantation window. Unlike peripheral NK cells, which are primarily cytotoxic, uNK cells exhibit low cytotoxicity and a high capacity for secretion of biologically relevant factors. They produce cytokines, chemokines, and angiogenic factors such as vascular endothelial growth factor (VEGF) [80–82], placental growth factor (PLGF) [83], and angiopoietins [84], which support spiral artery remodeling and placental vascularization [85]. Their proliferation and differentiation are driven by the decidual microenvironment, particularly through local cytokines like IL-15 secreted by stromal cells [86].

A critical aspect of trophoblast-uNK interactions is the recognition of fetal antigens. EVTs express non-classical MHC class I molecules, including HLA-G, and HLA-C, but lack HLA-A and HLA-B to minimize T cell activation [87]. HLA-G interacts with inhibitory receptors like killer immunoglobulin-like receptorn (KIR) 2DL4 on uNK cells, promoting tolerance by inducing cytokine secretion rather than cytotoxic responses. Additionally, soluble forms of HLA-G, such as HLA-G5, induce generation of “suppressor” macrophages that produce IL-10 and promote Treg generation [88]. HLA-C engages with KIRs on uNK cells, and specific KIR-HLA-C combinations influence placentation outcomes [89, 90]. For example, certain maternal KIR genotypes paired with fetal HLA-C variants can modulate the depth of trophoblast invasion, balancing tolerance and vascular support. uNK cells cluster around invading EVTs and spiral arteries, facilitating trophoblast migration and endothelial cell apoptosis to transform vessels into high-capacity conduits [91, 92]. Part of the ability of uNK cells in modulating trophoblast invasion is associated with production of interferon-gamma (IFN-γ), which regulates EVT differentiation and enhances matrix metalloproteinase (MMP) activity, aiding extracellular matrix degradation [93]. In vitro studies demonstrate that uNK-derived factors promote EVT outgrowth and migration, underscoring their regulatory role [94, 95]. Disruptions in uNK function, such as altered cytokine production, can impair implantation, while other studies have shown healthy uNK cells promote fetal growth [96].

Beyond uNK cells, decidual macrophages adopt an M2-like phenotype, characterized by anti-inflammatory properties [97]. These macrophages secrete cytokines such as IL-10 and transforming growth factor-beta (TGF-β), which suppress excessive inflammation and support tissue repair [98, 99]. They also clear apoptotic debris generated during trophoblast invasion, preventing the activation of autoimmune responses. Macrophages interact with uNK cells through cytokine crosstalk, reinforcing the tolerogenic environment. Interestingly, progesterone treatment decreases immunologically mediated miscarriage in part by enhancing tolerogenic properties of decidual macrophages [100, 101].

Treg cells, marked by FoxP3 expression, expand significantly in early pregnancy, constituting 10–20% of decidual CD4+ T cells [102]. Tregs suppress effector T cell responses against paternal antigens through both contact-dependent mechanisms (e.g., CTLA-4) and soluble factors like IL-10 and TGF-β [103]. Their absence or dysfunction in experimental models leads to fetal rejection, emphasizing their role in maintaining immune tolerance [104].

Innate lymphoid cells (ILCs) and dendritic cells (DCs) further shape the decidual environment. ILC3s produce IL-22, which enhances epithelial integrity and supports trophoblast survival [105, 106]. Tolerogenic DCs present antigens in a manner that promotes Treg induction, bridging innate and adaptive immunity to reinforce tolerance. Interestingly, administration of pregnancy promoting factors such as progesterone increase tolerogenic activity of dendritic cells, promoting local immune suppression and successful pregnancy [107].

The cytokine profile during early pregnancy transitions from a Th1-dominant state, with low levels of IFN-γ and TNF-α to support implantation, to a Th2/Th3-dominant profile, characterized by IL-4, IL-10, and TGF-β, to maintain tolerance [108]. IL-15 sustains uNK cell survival and function [86, 109], while progesterone induces galectin-1 expression on trophoblasts, which inhibits T cell activation [110, 111]. Extracellular vesicles released by decidual stromal cells carry immunosuppressive molecules, further promoting tolerance and preventing immune overactivation [112, 113].

In conclusion, successful pregnancy is dependent on a well-coordinated series of events that relies on multiple immunological mechanisms. There is some redundancy, however, in situations in which multiple immunological mechanisms are malfunctioning, the end result is fetal loss. This is observed in the devastating clinical condition of recurrent pregnancy loss (RPL).

## Miscarriage-associated immunological abnormalities

RPL, defined as two or more consecutive miscarriages before 20 weeks of gestation, affects approximately 1–5% of women trying to conceive, causing significant emotional and physical distress [114]. While factors like genetics, anatomical issues, hormonal imbalances, and blood clotting disorders play a role, immunological abnormalities are a major driver, contributing to roughly half of cases where no other cause is found. These cases often stem from a disrupted balance at the maternal-fetal interface, where the mother’s immune system fails to tolerate the fetus, which carries both her own and paternal antigens [115]. This failure can trigger inflammation, impair implantation, or lead to fetal loss through autoimmune reactions, mismatched immune recognition, dysfunctional immune cells, or imbalanced inflammatory signals.

Below, we outline these immunological abnormalities, their mechanisms, and their implications for diagnosis and treatment, emphasizing the need for tailored approaches to support successful pregnancies.

Autoimmune issues are a key factor in RPL, affecting about one in five cases [116]. The most common condition is antiphospholipid syndrome (APS), where antibodies attack molecules involved in blood clotting, causing blood clots, inflammation, and placental damage. These antibodies activate immune cells that release harmful substances, disrupting blood vessel formation and nutrient delivery to the fetus, often resulting in miscarriage [117, 118]. Other autoimmune conditions, like those involving antibodies against the thyroid [119], can also raise miscarriage risk, even if thyroid function appears normal, by affecting the uterine lining’s ability to support a pregnancy [120]. Additional antibodies, such as those targeting sperm or nuclear components, can further fuel inflammation, damaging the placenta and embryo. These autoimmune processes create an environment of excessive inflammation and clotting [121], which can often be managed with treatments like low-dose aspirin and heparin [122]. In addition to stimulation of thrombosis, APS mediates pregnancy loss through induction of inflammation, which is evidenced by the therapeutic effects of TNF-alpha blockers in these patients [123, 124].

The behavior of immune cells at the maternal-fetal interface is critical in RPL. Uterine natural killer (uNK) cells, which normally support blood vessel growth and placental development, can become overactive or overly abundant in RPL, disrupting these processes [125, 126]. Similarly, Treg cells, which dampen immune responses to protect the fetus, are often reduced in number or function, allowing inflammation to dominate [127–131]. Meanwhile, other immune cells, like those promoting inflammation, become overly active, damaging the uterine environment. Macrophages and dendritic cells, which help balance immune responses, may also shift toward inflammatory states, worsening tissue damage and hindering implantation [132–139]. These cellular imbalances create a hostile environment for the embryo, preventing it from developing properly.

Imbalances in immune signaling molecules, known as cytokines, further contribute to RPL. In healthy pregnancies, anti-inflammatory signals dominate to support fetal growth, but in RPL, inflammatory cytokines predominate, promoting tissue damage and reducing the uterus’s ability to sustain a pregnancy [140]. Reduced levels of protective molecules weaken immune tolerance, while increased inflammatory signals can trigger placental damage or impair embryo implantation [141].

## Immune modulatory interventions with clinical signals of efficacy

In patients with APS treat,ment consists of a combination of low-dose aspirin (LDA) and low molecular weight heparin (LMWH), typically given as a daily injection. Aspirin reduces clotting and inflammation, while heparin prevents blood clots and calms immune overactivity. This regimen can boost live birth rates to 70–80% in women with APS, compared to much lower rates without treatment [142]. Treatment often starts before conception and continues into late pregnancy, significantly cutting miscarriage risk. However, this approach is most effective for confirmed APS cases; in women without clear autoimmune markers, the benefits are less certain, and risks like bleeding can arise, making careful patient selection critical [143].

Another intervention, intravenous immunoglobulin (IVIG), is used for RPL linked to abnormal immune cell activity, such as overactive natural killer (NK) cells or reduced regulatory T cells, which normally suppress harmful immune responses [144, 145]. IVIG, made from pooled human antibodies, works by neutralizing harmful antibodies, calming inflammation, and balancing immune cell function to prevent the mother’s body from rejecting the fetus [16]. Administered every few weeks starting before or early in pregnancy, IVIG has shown success in specific cases, raising live birth rates to 80% in women with immune cell irregularities, compared to 40–60% without treatment [146, 147]. However, its high cost, potential side effects like allergic reactions, and inconsistent results in broader RPL cases limit its routine use. It’s most effective when tailored to patients with clear signs of immune dysregulation, highlighting the importance of thorough immune testing before treatment.

Corticosteroids, such as prednisone, are sometimes used to reduce inflammation in RPL cases tied to autoimmune issues like thyroid disorders or chronic uterine inflammation [148]. These drugs lower inflammatory signals, calm overactive immune cells, and support the uterine environment for implantation. When combined with aspirin or progesterone, low-dose prednisone can improve live birth rates significantly, especially in women with autoimmune thyroid conditions, reducing miscarriage risk by up to half [149]. However, long-term use carries risks like hyperglycemia, fluid retention, and blood pressure, elevation, so treatment is typically short-term and carefully monitored. In cases without clear autoimmune drivers, corticosteroids are less effective, and guidelines caution against their routine use due to potential harm to both mother and fetus.

Another therapy is leukocyte immunization, where paternal or donor immune cells are injected to “train” the mother’s immune system to tolerate the fetus [150]. Paternal lymphocyte immunization, also known as lymphocyte immunization therapy (LIT) or paternal lymphocyte immunotherapy, is an immunomodulatory treatment used for couples experiencing RPL [151]. The procedure involves collecting a blood sample from the prospective father, isolating his lymphocytes processing them, and then injecting them, typically intradermally or intravenously, into the prospective mother. The goal is to induce maternal immune tolerance to paternal antigens present in the embryo or fetus, which carries half its genetic material from the father. By exposing the mother’s immune system to paternal cells in advance, the therapy aims to promote protective antibodies, shift immune balance (such as improving Th1/Th2 ratios or increasing Treg cells), and reduce natural killer cell activity or other rejection mechanisms [152]. While early studies suggest benefits, inconsistent results and risks such as immune reactions, have reduced its use. Similarly, drugs targeting specific inflammatory molecules, like anti-TNF agents [153, 154], showed early promise in calming excessive inflammation in RPL. However at least one study reported increased pregnancy complications after TNF-alpha blockade in a pregnant woman suffering from inflammatory bowel disease [155, 156].

Mesenchymal stem cells (MSCs) may offer a unique, safe and effective means of overcoming RPL, alone or in combination with existing modalities. Existing RPL therapies such as aspirin, LMWH, IVIG exert a defined anti-inflammatory effect which is based on the amount of agent administered but not necessarily on biological need. Our proposed concept of using MSC for RPL is rooted in the unique “living therapeutic” characteristics of these cells including: a) ability to produce corresponding amount of anti-inflammatory agents based on extent of existing inflammation [69]; b) ability to produce angiogenic cytokines that could potentially assist in placental growth [157]; and c) promising data from preclinical studies [67, 158–167].

## Rationale for MSC to reduce recurrent pregnancy loss

We propose the initial utilization of MSCs as immune modulatory therapy for woman suffering from RPL caused by immunological factors outside of APS. MSCs are versatile adult stem cells from sources like bone marrow, fat tissue, umbilical cord, amniotic fluid and membrane, and menstrual blood, offering a promising new approach to suppressing pathological inflammation while stimulating angiogenesis and placental growth. With their ability to modulate immunity, reduce inflammation, and repair tissue, MSCs could transform RPL treatment by creating a supportive environment for pregnancy. Here, we explain why MSCs are uniquely suited to address RPL, focusing on their mechanisms, potential benefits, and future promise.

The core strength of MSCs in treating RPL lies in their ability to balance the maternal immune system, which often becomes overactive in RPL, attacking the fetus as if it were a foreign invader. In a healthy pregnancy, the differentiated endometrium, called the decidua, creates a tolerant environment through immune cells and signaling molecules that protects the fetus while supporting placental growth. In RPL, this balance tips toward inflammation, with harmful immune cells like cytotoxic natural killer (NK) cells and inflammatory molecules overpowering protective ones. MSCs counteract pathological immunity by releasing anti-inflammatory substances that calm aggressive immune responses and promote protective cells, such as Treg cells, which may decrease the risk of immunologic rejection of the fetus. The fundamental importance of Treg in maintaining pregnancy was shown in a study by Shima et al. in which antibody mediated depletion of these cells resulted in increased fetal loss in allogeneic but not syngeneic pregnancies [168].

Allograft protective effects of donor, recipient, as well as third party MSCs have been demonstrated in models of kidney [169–171], lung [172–176], and heart [177] transplantation. Additionally, MSCs have been shown to protect against numerous major autoimmune conditions including rheumatoid arthritis [178], multiple sclerosis [179], and systemic lupus erythematosus [180].

MSCs also shift immune cells in the uterus toward a more tolerogenic state, reducing tissue damage and fostering a nurturing environment for the embryo. In animal studies mimicking RPL, MSCs have prevented miscarriage by lowering inflammation, boosting protective signals, and reducing harmful immune cell activity [67, 158–161]. This ability to restore immune harmony directly targets the root of many unexplained RPL cases, offering a more precise solution than broad immune-suppressing drugs.

Beyond dampening pathological immunity, MSCs excel at repairing and regenerating the uterine lining, which is critical for women with RPL caused by conditions like thin endometrium, scar tissue, or chronic inflammation. A healthy endometrium must thicken and develop blood vessels to support embryo implantation and growth, but in RPL, immunologically mediated damage or poor tissue quality often prevents this. MSCs can transform into endometrial cells or release growth factors that stimulate blood vessel formation and tissue repair, improving the uterus’s ability to sustain a pregnancy [157]. In women with conditions like Asherman syndrome, where scar tissue hinders implantation, MSCs have restored endometrial thickness and function, leading to better pregnancy outcomes. For example, in cases where the uterine lining is too thin, often linked to higher miscarriage rates, MSCs have increased thickness enough to support implantation [181].

In animal models designed to mimic RPL, MSCs have consistently reduced miscarriage rates by balancing immune responses and repairing uterine tissue. These studies show MSCs lowering harmful inflammation, enhancing protective immune cells, and improving placental development, leading to healthier pregnancies [67, 158, 159, 161]. It is believed that exosomes derived from MSC exert similar biological properties to the cells themselves in terms of tolerogenic activities such as treatment of murine models of multiple sclerosis [182–184], rheumatoid arthritis [185, 186], or type 1 diabetes [187]. Accordingly, one study did demonstrate that MSC exosomes are capable of reducing fetal loss in the CBA/J x DBA/2 model [162].

In small human studies, particularly for women with uterine scarring or thin endometrium MSCs have improved uterine health, with some women achieving pregnancies and live births after treatment [188]. For instance, transplanting MSCs into the uterus has thickened the lining and restored menstrual function, paving the way for successful pregnancies in women previously unable to conceive [189].

MSCs offer practical advantages that make them an appealing therapy. They can be obtained non-invasively from sources such as umbilical cord or menstrual blood, avoiding ethical concerns tied to other stem cell types. Unlike embryonic stem cells, MSCs have a low risk of forming tumors, and their low immunogenicity means they can often be used from donors without triggering rejection. For women with RPL, autologous MSCs from their own fat or menstrual blood eliminate that risk. MSC treatments can be delivered through simple methods such as infusion into the uterus or bloodstream, often in outpatient settings, reducing the burden compared to repeated fertility procedures. By addressing both immune and tissue issues, MSCs could decrease reliance on costly and invasive fertility treatments, offering a less stressful path to pregnancy. An additional benefit of MSC may be in their potential to reduce pre-eclampsia, which is mediated in part by poor placental blood vessel formation. The high angiogeneic activity of MSC may conceptually be beneficial in this patient population [190, 191]. Their potential to prevent future losses by improving overall uterine health adds to their appeal for long-term reproductive success.

## Conclusion

Successful pregnancy requires a finely tuned immune environment in the decidua, where uNK cells predominate and support spiral artery remodeling, trophoblast invasion, and placental development while maintaining low cytotoxicity. In RPL, dysregulation often manifests as increased uNK cell numbers or activity, reduced Treg suppression of effector cells, and imbalances favoring inflammatory pathways (e.g., IFN-γ and IL-17 elevation). These abnormalities disrupt vascular adaptation and immune homeostasis, leading to inadequate placentation and fetal rejection-like responses.

We propose the use of MSCs as a therapeutic option for RPL. These cells have demonstrated clinical efficacy in chronic, as well as severe immune dysregulation states. In December 2024, the U.S. Food and Drug Administration (FDA) approved the first MSC-based therapy, Ryoncil (remestemcel-Lrnd), an allogeneic, bone marrow-derived MSC product), for steroid-refractory acute graft-versus-host disease (SR-aGVHD) in pediatric patients aged 2 months and older [71]. GVHD represents an extreme form of unrestrained alloimmune reactivity, involving hyperactivated T cells, NK dysregulation, and cytokine storms pathophysiological features that parallel, albeit in exaggerated form, the immune aberrations observed in unexplained RPL. The approval of Ryoncil underscores the therapeutic potential of MSCs to re-establish immune homeostasis in conditions of profound dysregulation.

Given the shared immunological underpinnings between GVHD and RPL, particularly NK cell hyperactivity and deficient Treg-mediated tolerance and the established mechanisms by which MSCs correct NK abnormalities, promote Treg expansion and function, suppress pro-inflammatory cytokines, and restore decidual immune balance, we propose that MSC-based therapies warrant investigation as a novel treatment modality for unexplained RPL. There are still areas of development that need to be addressed before MSC based therapies for RPL can become a clinical reality, these include: a) dose optimization; b) selection of MSC type with most immune modulatory potential; c) selection of optimized patient population; and d) full clinical evaluation.

**Fig. 1:**
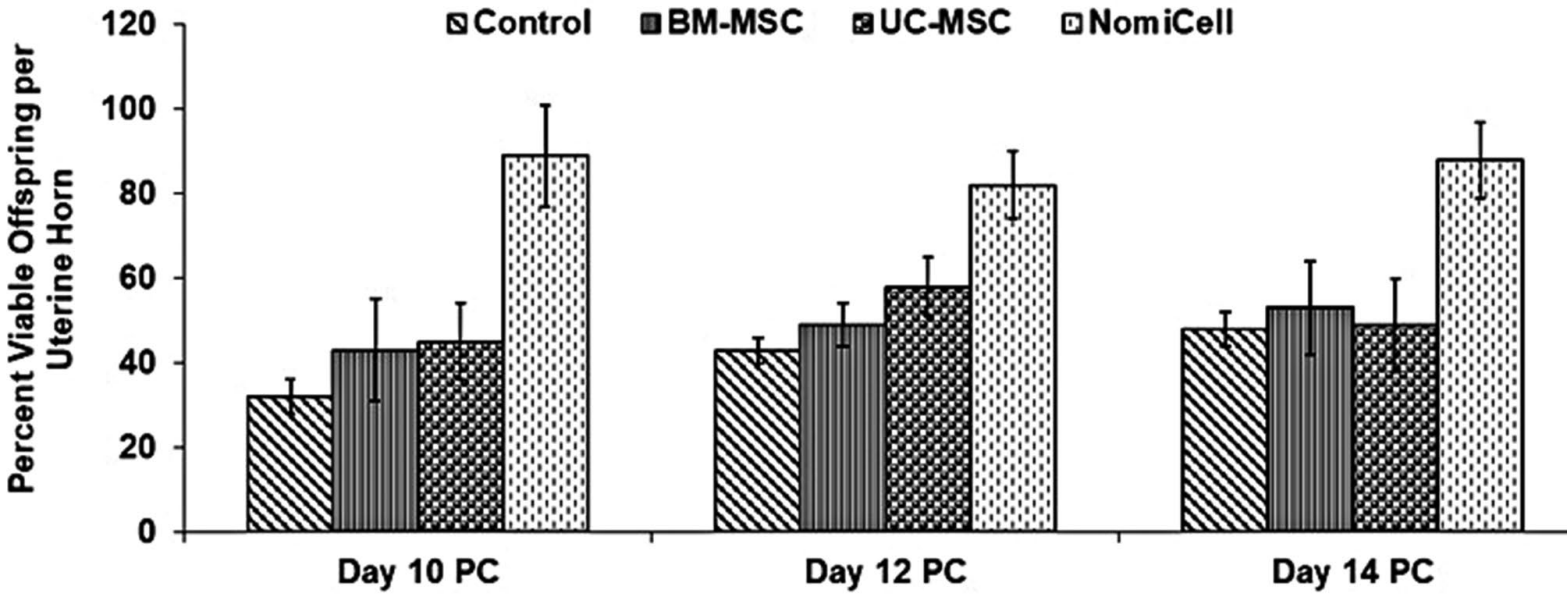
Reduction of RPL by NomiCell™ administration: CBA/J females were mated with DBA/2 males at Day 0 post ciotum (PC). Control saline, or bone marrow (BM), umbilical cord (UC) mesenchymal stem cells (MSC) or NomiCell™ were administered at day 1, 3, and 5 PC intravenously at a concentration of 500,000 cells. Assessment of fetal viability was performed by a blinded observer. Ten mice per group were utilized

In order to develop a commercial product that can enter preclinical and eventual clinical testing, our company, Aureum Therapeutics Inc has been developing NomiCells™, an umbilical cord MSC based product which is optimized for enhanced immune modulatory activity based on ability to secrete interleukin-10. Preliminary data by our group has demonstrated that administration of this novel subset of MSC to the CBA/J X DBA/2 model of RPL results in enhanced pregnancy in a superior manner to control umbilical cord derived, as well as bone marrow derived MSC (Fig. 1). We are continuing research in this area with the idea of commercializing a natural cell-based intervention for RPL and immunologically mediated pregnancy complications.

## Data Availability

All relevant data analyzed during this study are included in this published article.
